# A role for microbial selection in frescoes’ deterioration in *Tomba degli Scudi* in Tarquinia, Italy

**DOI:** 10.1038/s41598-017-06169-0

**Published:** 2017-07-20

**Authors:** Maria Cristina Tomassetti, Angela Cirigliano, Chiara Arrighi, Rodolfo Negri, Francesco Mura, Maria Lorella Maneschi, Maria Donatella Gentili, Mariarita Stirpe, Cristina Mazzoni, Teresa Rinaldi

**Affiliations:** 1 Freelance restorer, Via Flavia 16, 00062 Bracciano Rome, Italy; 2 La Sapienza University of Rome, Departement of Biology and Biotechnology “Charles Darwin”, Piazzale Aldo Moro, 5, 00185 Rome, Italy; 3 Freelance restorer, Via San Maria Mediatrice 10, 00165 Rome, Italy; 4 La Sapienza University of Rome, Departement of Chemistry, Piazzale Aldo Moro, 5, 00185 Rome, Italy; 5 Archaeologist, Presidente FAI, Delegazione Viterbo, Via XX Settembre 56, 01016 Tarquinia, Italy; 6 Archaeologist, Via de Carolis 135, 00136 Rome, Italy

## Abstract

Mural paintings in the hypogeal environment of the *Tomba degli Scudi* in Tarquinia, Italy, show a quite dramatic condition: the plaster mortar lost his cohesion and a white layer coating is spread over almost all the wall surfaces. The aim of this research is to verify if the activity of microorganisms could be one of the main causes of deterioration and if the adopted countermeasures (conventional biocide treatments) are sufficient to stop it. A biocide treatment of the whole environment has been carried out before the conservative intervention and the tomb has been closed for one month. When the tomb was opened again, we sampled the microorganisms present on the frescoes and we identified four *Bacillus* species and one mould survived to the biocide treatment. These organisms are able to produce spores, a highly resistant biological form, which has permitted the survival despite the biocide treatment. We show that these *Bacillus* strains are able to produce calcium carbonate and could be responsible for the white deposition that was damaging and covering the entire surface of the frescoes. Our results confirm that the sanitation intervention is non always resolutive and could even be deleterious in selecting harmful microbial communities.

## Introduction

**Figure 1:**
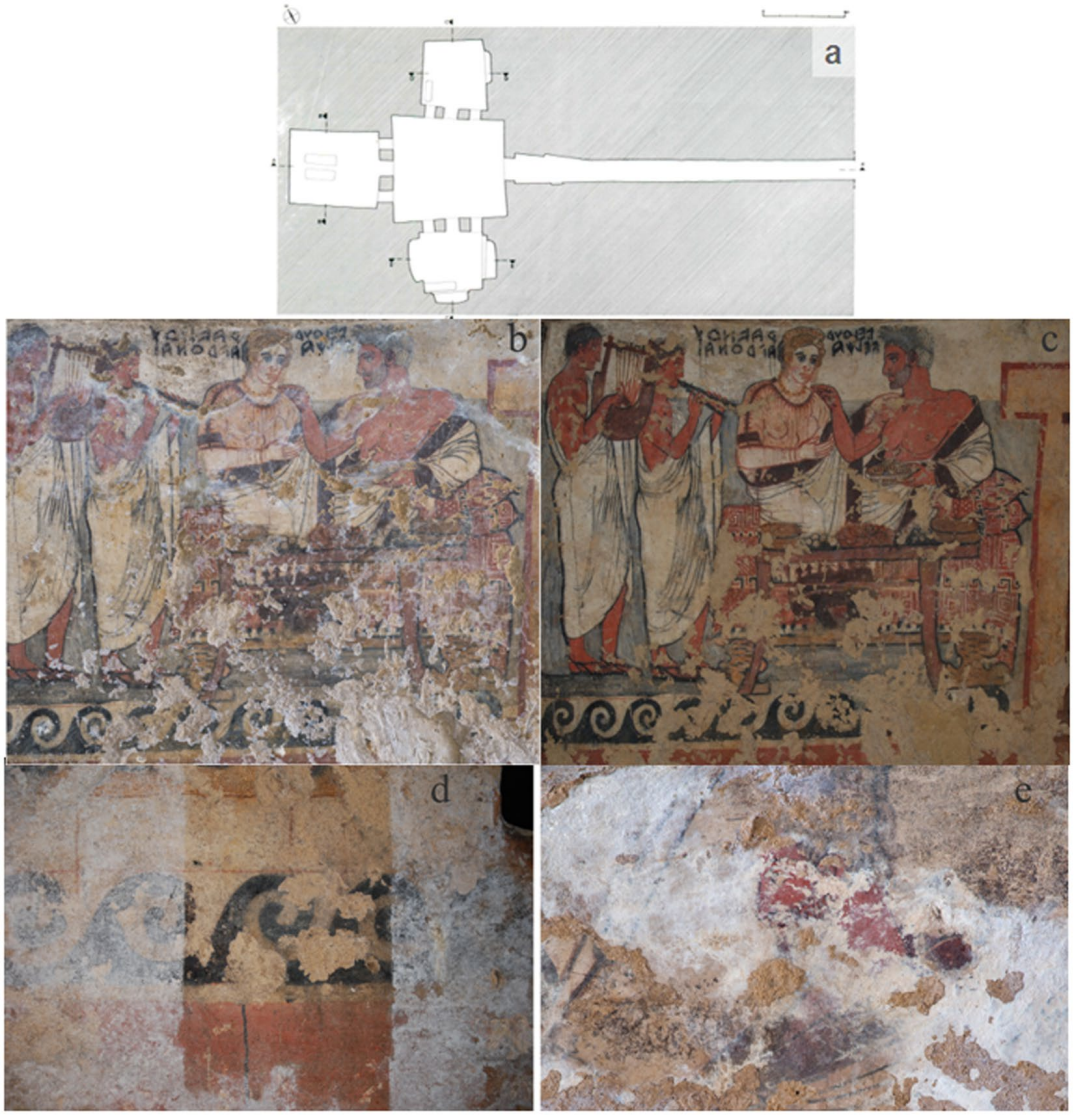
*Tomba degli Scudi*, Tarquinia, Italy. Intervention of the restorers to eliminate the white deposition (CaCO_3_) which covers the walls. (**a**) Hand drawn map of the tomb from Maria Cristina Tomassetti, based on measures taken during the intervention of restoration. The restoration presented in this paper is referred to the central chamber; bar, 5 meter. (**b**) Northern wall before the intervention. (**c**) Northern wall after the elimination of CaCO_3_; it is possible now read the names Ravnthu Aprthnai, on the left and Velthur Velcha, on the right. (**d**) Western wall, in the central part of the fresco the white deposition was eliminated as a tester for the restorers. (**e**) The thick patina in the eastern wall.

The *Tomba degli Scudi* is located in Monterozzi necropolis of Tarquinia, UNESCO site since 2004. Dated to the Late Classical period (mid IV century B.C.) and rediscovered in 1870, is decorated with important frescoes that are examples of the figurative programs of the great aristocratic tombs of that era^1^. It is one of the largest tombs of Tarquinia and its structure simulates an Etruscan house with a central atrium and three chambers (Fig. 1a). The ceiling of the atrium is gabled, with a painted decoration that simulates wooden beams^2, 3^. The frieze with the shields in the rear chamber, that gives name to the tomb, probably highlights the predominant role of the aristocratic family in the military field. It celebrates the virtues and the rank of the Velcha family, immortalising the moment of departure of the deceased to the afterlife and the funeral banquet ideally involving all family members with the founder of the tomb, Larth Velcha and his wife Velia Seithiti, and his parents Velthur Velcha and Ravnthu Aprthnai (Fig. 1b before restoration, 1c, after restoration). In the central and in the rear chamber several important inscriptions are painted, mainly referring to the powerful Tarquinian *gens* Velcha^4^. At the end of the restoration the paintings and the inscriptions of the tomb will be submitted to a critical revision.

The paintings are in *fresco* technique, by applying natural pigments dissolved in water (iron oxides for yellow and red, lime for the white and black coal) directly on the wet plaster, composed by calcium hydroxide and grounded *macco* (a organogenic limestone rich of small shells). When the tomb is closed, the environmental conditions are those typical of an isolated hypogeal site. The microclimate is rather stable, with temperatures ranging from 15 °C to 17 °C and relative humidity between 95 and 100%, which can lead to reach the dew-point temperature, with water condensation on surfaces.

When the *Soprintendenza Archeologia, Belle Arti e Paesaggio* decided to restore the frescoes, walls were covered of a white deposition corresponding to calcium carbonate (CaCO_3_, calcite), (Fig. 1b,d,e). In same part of the fresco the aspect and the consistency of this white layer did not resemble a typical carbonate saline efflorescence, hence the need of a biological investigation to detect the nature of the *patina*. Two approaches can be used to investigate the relationship between microorganisms and deterioration of the frescoes. One has recently emerged with the development of Next Generation Sequencing (NGS) techniques which opened up perspectives of wide-range metagenomic analyses. Thanks to the rapid development of this methodology, it is possible to have a screenshot picture of all the microorganisms present on a wall of an archeological site. From an environmental sample both DNA or RNA can be extracted and sequenced. In particular, RNA sequencing has the potential to identify metabolically active microorganisms. This approach was successful applied in another etruscan tomb^5^. The second approach is a classical microbiological study which first identifies on plates microorganisms sampled on site and then DNA sequence is performed from pure cultures to unequivocally classify them. Here, we used the latter approach for two reasons: first, when the restorers were contacted by the *Soprintendenza Archeologia, Belle Arti e Paesaggio*, a preventive sanitation had already been performed with ammonium quaternary salts (Preventol) and the tomb remained sealed for one month. A full metagenomic analysis at this point would have been much less informative. Second, we performed this study to support the work of restorers who were interested in understanding if microorganisms were responsible for the calcite deposition and if they survived to the sanitation treatment. At this purpose it was important to show that some of the *Bacillus* species, which we isolated, were indeed capable to produce calcite.

## Results

### Isolation of microorganisms

For this study, we decided to proceed with a classical microbiological isolation techniques and we did not use a metagenomic approach, since our aim was to find microorganisms able to survive to the biocide treatment by producing biofilms and/or spores; moreover, it is known that the yield of DNA extracted in presence of a coat of spores could be below the limit required for a miningful metagenomic analysis performed by 16S rDNA amplification^6^. Before restoration of the *Tomba degli Scudi*, a sanitation treatment was done; for technical reasons, we were not able to sample microorganisms before the sanitation, but the restorers were the first to enter in the tomb after one month of biocide treatment and collected the samples from the frescoes before any intervention in the tomb.

At the same time, a qualitative control was performed on air and ground samples. Qualitative results from air show a low variety of microorganisms inside the tomb: in LB medium, from outdoor, a great variety of bacteria were grown, while only moulds were sampled in LB indoor (Supplementary Fig. S1). From samples collected in the ground, the qualitative observation of LB plates showed a predominance of *Bacillus* species indoor, if compared with a greater variability of bacterial species sampled outdoor (Supplementary Fig. S2). We conclude that the biocide treatment was effective on the majority of bacteria present in the tomb, but that was ineffective on microorganisms able to produce resistant forms of life, such as moulds and spore forming bacteria.

**Figure 2:**
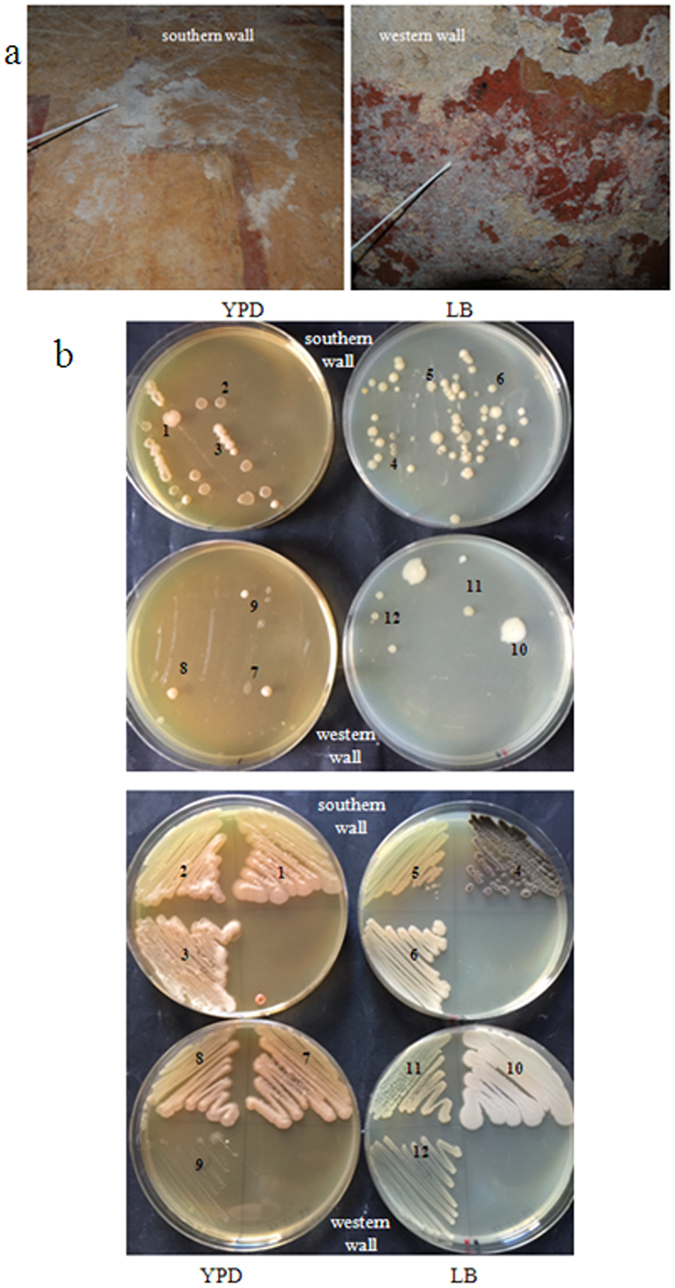
Microorganisms isolated from the walls. (**a**) Samples from southern and western walls were taken with two sterile swabs and (**b**) streaked in YPD and LB plates. (**c**) After colony morphology and microscopic observations, 12 pure cultures were chosen for further analysis.

**Figure 3:**
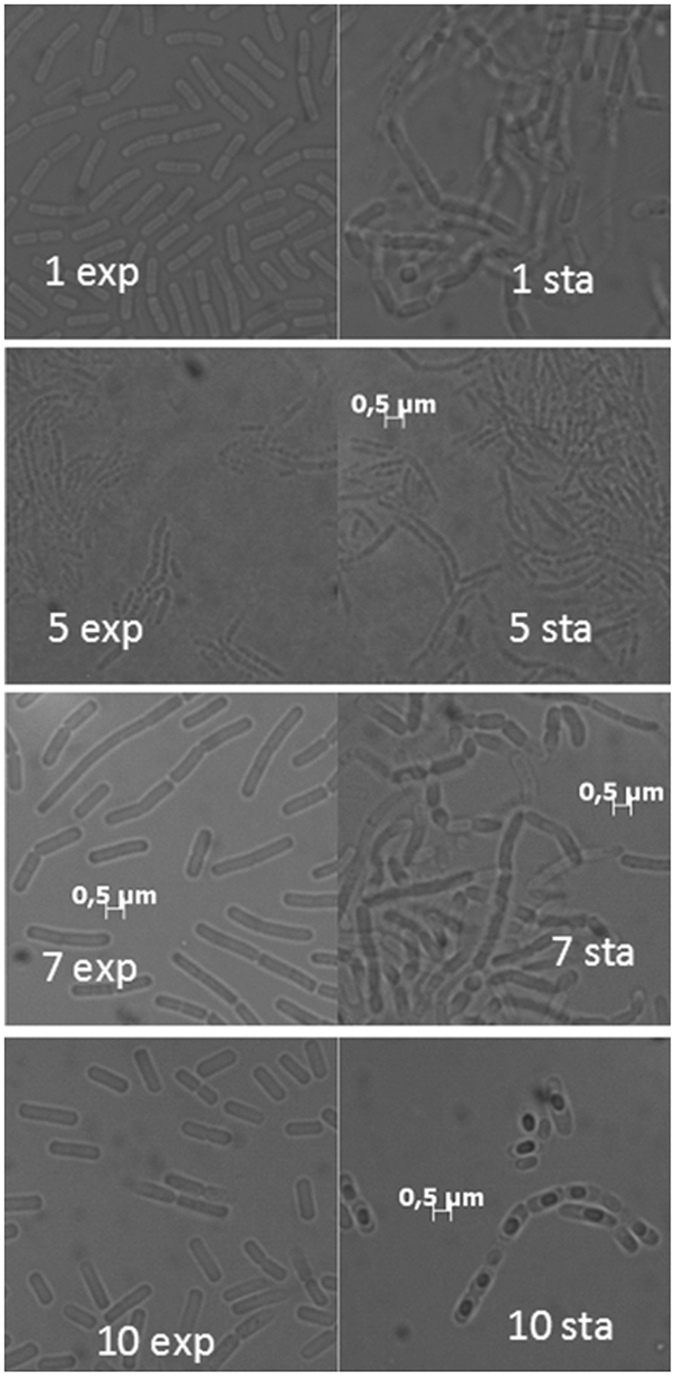
Bacterial morphologies of the four *Bacillus* species (samples 1, 5, 7 and 10) in exponential (exp) and stationary phase (sta). The pure cultures were grown in complete medium over night (exp) and three days (sta) at 28 °C. Microscopic analysis shows bacteria of the Genus *Bacillus*. Bar: 0,5 micrometry.

A white colonisation was uniformly present on the surface of the frescoes, so samples were taken from the southern and western wall and (Fig. 2a) and streaked in YPD and LB plates (Fig. 2b); 12 colonies with macroscopically different morphologies were chosen for further analysis. The 12 pure cultures (Fig. 2c) were observed at the microscope; the sample 4 was a mould and it wasn’t further investigated in this work. From the remaining 11 cultures, we identify 4 different species present on the white deposition on the frescoes (Fig. 3). In stationary phase, the bacterial strain 1, 5 and 7 produced filaments (spores were also observed at the microscope), while culture 10 produced 100% of spores. These results indicated that the biocide treatment has left alive microorganisms forming filaments like biofilms and spores (bacteria and mould).

### Identification of *Bacillus* species

In order to identify the bacterial species the 16S rDNA was amplified after extraction from pure cultures and sequenced (see Materials and Methods).

**Figure 4:**
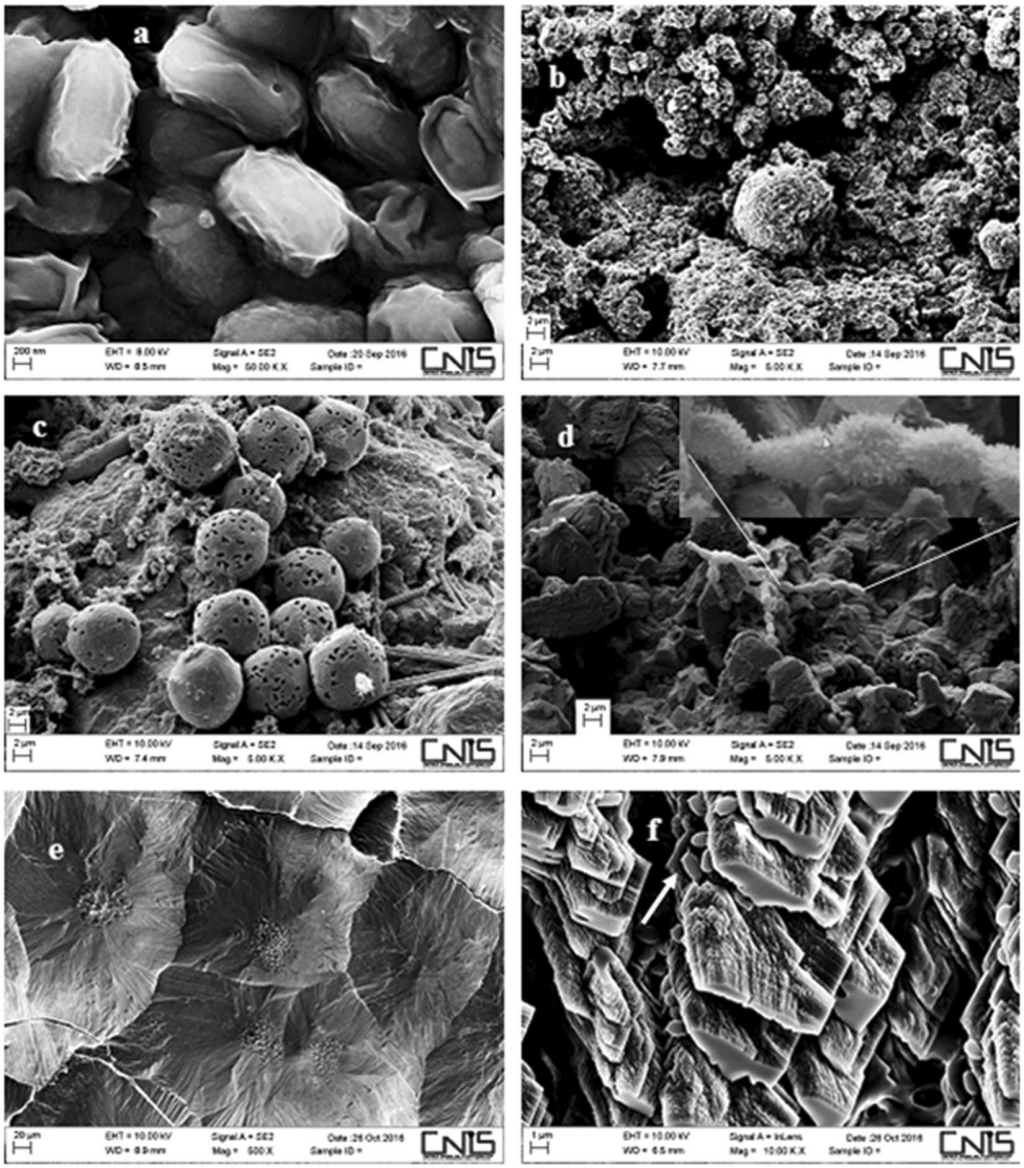
Scanning electron micrographs (SEM) of samples from the *Tomba degli Scudi*. (**a**) Purified spores from *Bacillus cereus* (sample 10) isolated from the white deposition of the western wall. (**b**) Spheroidal element from the western wall covered of white deposition (see also Fig. 1d) corresponding to CaCO_3_. (**c**) Spores of a mould from the thick white deposition of the eastern wall (see Fig. 1e). (**d**) A microorganism in the thick deposition in the eastern wall is producing CaCO_3_ (see Figure S4b). (**e**) CaCO_3_ produced by a pure culture of sample 7 grown on YPD supplemented with urea (see also Figure S6). (**f**) CaCO_3_ produced by a pure culture of sample 10 in YPD supplemented with urea; white arrows indicate the spores of this microorganism mixed with the CaCO_3_.

Table S1 shows the closest relatives *Bacillus* species of the *Bacillus* isolates in the *Tomba degli Scudi*. From the online BLAST (https://blast.ncbi.nlm.nih.gov/Blast.cgi?PROGRAM=blastn&PAGE_TYPE=BlastSearch&LINK_LOC=blasthome) we identified as the closest bacterial relatives of sample 1 *Bacillus [Brevibacterium] frigoritolerans* and *Bacillus simplex* strains; of sample 5 *Fictibacillus barbaricus* and *Fictibacillus phosphorivorans* strains; of sample 7 *Brevibacterium frigoritolerans* and *Bacillus simplex* strains; of sample 10 *Bacillus cereus* strain. Figure 4a shows the purified spores from the sample 10 isolated in the western wall. Filogenetic analysis is showed in Figure S3.

### Biodeterioration of frescoes by *Bacillus* species

It has been already demonstrated that bacterial biofilm production positively correlates with calcite deposition^7^, so in order to identify the composition of the white deposition on the walls of the tomb (Fig. 1c), a sample of the western wall was analysed by scanning electron microscopy (SEM). The atomic composition (see Fig. S4a) indeed suggested the presence of calcite (CaCO_3_), see the spherical deposition in Fig. 4b. The thick white deposition of the eastern wall (Fig. 1e) was also analysed by SEM and was identified as calcite (not shown); from this analysis also mould spores and a bacterium covered of a coat of CaCO_3_ (Fig. 4c and d, respectively) were identified (see also Fig. S4b). It is well established that calcium carbonate crystals are a byproduct of bacterial metabolism, such as urea hydrolysis performed by the urease enzyme^6, 8^. Thus, we tested the capability of the isolated *Bacillus* species to depose calcium carbonate in YPD supplemented with urea (Fig. S5)^7^. Samples 1 and 7 were able to depose calcium carbonate crystals, sample 10 produced crystals in lower amount, while sample 5 didn’t depose calcium carbonate. One of the closest relative bacterium of sample 5, *Fictibacillus barbaricus* was described as urease negative, which is in line with the absence of calcite deposition in our sample 5 in presence of urea (Fig. S5). This organism was isolated for the first time as *Bacillus barbaricus*, in a project concerning bacterial colonization on a experimental wall painting^9^, and later renamed *Fictibacillus barbaricus* ^10^. The YPD plates supplemented with urea with our pure cultures were observed at the stereomicroscope, samples 1 and 7 had the same type of crystals, while sample 10 showed different depositions. We analysed the depositions from samples 7 and 10 with SEM. The crystals (Fig. 4e,f) corresponded to an inorganic compound corresponding to CaCO_3_. This result demonstrated that microorganisms isolated from the walls in the tomb were able to depose calcite and possibly responsible for producing the layer, which covers completely the frescoes.

## Discussion

The aim of this work was to identify the microorganisms which survived to the biocide treatment in the *Tomba degli Scudi*, in order to verify their potential role in bio-deterioration of frescoes. Despite the etruscan frescoes consist of one of the most important Italian artistic heritage, a few works were published on bio-deterioration^5, 11^ and most studies of microorganisms responsible for biodeterioration were oriented to biotechnological applications^12–14^.

The value of these cultural heritages is inestimable, however, every year the expenditure for decontamination in museums and for artworks is very high, hence the need to find the best solution for restoration cheap and durable. The most challenging aspect of biocide treatment is that the frescoes are infested by a mixture of microorganisms with different sensibility to the applied chemical compounds. From the results of our work and others^5^, this type of treatment is not resolutive and could even further compromise the state of the frescoes by selecting more detrimental bacterial populations. Our results indicated that after a month of biocide treatment the majority of microorganisms were killed, while remained alive organisms capable of producing resistant forms of life, namely *Bacillus* species and moulds. Indeed, *Bacillus* species are abundant in isolates of artworks^5, 6, 9, 11, 15, 16^. In the specific case of *Tomba degli Scudi*, three out of four isolated *Bacillus* species were able to depose CaCO_3._ Recently, another strain of *B. cereus* was described to be able to produce calcite^17^. This is a bad news, since in particular the spores from the *Bacillus cereus* group, to which *Bacillus anthracis* belongs to, are the most recalcitrant to decontamination. We conclude that the biocide treatment is useless and even detrimental because, after the sanitation, particular bacteria or fungi are in the condition to proliferate on the frescoes. This secondary colonisation relies on different factors and it is specific for each tomb. Indeed, the intervention to solve the problem of deterioration of frescoes should be carefully assessed with an interdisciplinary approach, involving biologist, chemists and geologists, since each site has a delicate equilibrium of microflora and environment. First, in the hypogeal tombs thermohygrometric values should be monitored to control humidity. Second, the visitors should be reduced and the time of stay in the tomb should be very short, since the carbon dioxide emitted through breathing could accelerate the development of microorganism; in fact, bacteria could perform a CO_2_ uptake from the atmosphere, dissolve the rock and produce CaCO_3_^6^. Third, the type of illumination (if it has to be used) should be carefully chosen because it could accelerate and select the growth of cyanobacteria: as an example, in the Catacombs of St. Callistus, the lights present in the site had enhanced the growth of *Actinobacteria* ^18^ and to try to solve the problem, a study was performed to induce stress to microorganisms using monochromatic blue light for 10 years. The results revealed that *Actinobacteria* were sensibly reduced, but at the advantage of the growth of *Proteobacteria* and *Firmicutes*. These results underline again the essential problem of secondary microorganisms selection during the restoration of frescoes: of interest, among Firmicutes, *Bacillus* species (*B. cereus*, *B. simplex* and *B. megaterium*) increased in the Catacombs of St. Callistus^19^. In another study, in the etruscan tomb of the Ducks at Veio, the biocide treatment destroyed the original actinomycetes and bacterial populations and selected more resistant and detrimental fungal species^20^.

Indeed, *Bacillus* spores are highly resistant to environmental stresses and moreover spores of *Bacillus, Streptomyces* and mould species, could be deposited again on the surfaces from air, leading to a new colonisation.

We confirm that a general sanitation is useless and alters the equilibrium of the microorganism community, often at the advantage of spore producing resistant strains. In the future, in order to avoid the alteration of the tomb environment, in association with the measures described above, we suggest periodical microbiological investigation to monitor the state of the tomb and eventually periodical cycles of frescoes’ cleaning. Finally, a “personalized” sanitation that targets microorganism populations, specifically detected on the site and potentially harmful, could be taken in consideration to remedy to previous interventions.

## Methods

### Samples collection

Samples were taken after the sanitation procedures. Air: plates with complete medium YPD (rich medium containing 1% Bacto-peptone, 1% yeast extract, 2% glucose), YPD + 80 micrograms/ml geneticine, LB (0,5% Yeast Extract, 1% Bacto-Tryptone, 0,5% NaCl, 1 ml NaOH 1N), LB + 100 micrograms/ml ampicillin were opened for 15 minutes in the tombs and outdoor (as a qualitative control). Ground: 1gr of ground from the floor of the tomb and from outside were put in 1 ml of sterile water and 100 microliters were plated in LB medium. Frescoes: with sterile cotton swabs two samples from the southern and western walls were taken and streaked in sterile conditions on YPD and LB plates. The growth was followed for 2 weeks. The production of calcium carbonate was followed on YPD plates containing 3 gr/l of Urea and LB plates containing 25 g/l of CaCl_2_.

### Microscopy

Bacterial pictures were taken with a Zeiss Axio Imager Z1 Fluorescence Microscope with AxioVision 4.8 Digital Image Processing System, and the objective lens used was 63 × oil. Scanning electron microscopy (SEM) micrographs and the EDX spectra have been obtained using a FESEM Zeiss Auriga equipped with Bruker Quantax detector. For improving the samples’ conductivity, they have been coated with a 50 nm layer of gold (Fig. 4a–c) and chromium (Fig. 4d) using the Quorum Q150T ES sputter machine.

### Amplification, sequencing and analysis of 16S-rRNA gene

A single colony for each isolated was picked and suspended in 20 µl of microLYSIS buffer (Labogen, Rho, Italy). Total DNA was prepared according to the manufacturer’s instructions. 2 μl of DNA extracts were use for the amplification of 16S ribosomal DNA (rDNA) using standard PCR protocol by the universal bacterial primers P0 (5′ -GAGAGTTTGATCCTGGCT-3′) and P6 (5′CTACGGCTACCTTGTTAC-3′)^21^. 16S rDNA amplification was verified by agarose gel electrophoresis. The band of about 1500 bp was purified (Gel/PCR DNA fragments Extraction kit Geneaid cat. DF 100) and sequenced with both primers by a sequencing service (http://www.biofabresearch.it/index2.html). The resulting sequences were submitted to GenBank and received the accession numbers reported in Table S1. The resulting sequences were analyzed using the online BLAST program https://blast.ncbi.nlm.nih.gov/Blast.cgi?PROGRAM=blastn&PAGE_TYPE=BlastSearch&LINK_LOC=blasthome ^22^.

The online resource http://www.phylogeny.fr/simple_phylogeny.cgi was used to construct the filogenetic tree.

## Electronic supplementary material

supplementary material
