# Supplementary material for: A role for microbial selection in frescoes’ deterioration in Tomba degli Scudi in Tarquinia, Italy
Source: Sci Rep. 2017 Jul 20;7:6027. doi: 10.1038/s41598-017-06169-0 (PMC5519700; doi:10.1038/s41598-017-06169-0)
Supplement: Supplementary file 1 — supplementary material [file 41598_2017_6169_MOESM1_ESM.pdf]

Supplementary informations

A role for microbial selection in frescoes' deterioration in *Tomba degli Scudi* in Tarquinia, Italy

Maria Cristina Tomassetti[1], Angela Cirigliano[2], Chiara Arrighi[1,] Rodolfo Negri[2], Francesco Mura[3], Maria Lorella Maneschi[4], Maria Donatella Gentili[5], Mariarita Stirpe[2], Cristina Mazzoni[2] and Teresa Rinaldi[2]*

[1] Freelance restorer
[2] La Sapienza University of Rome, Dept. of Biology and Biotechnology "Charles Darwin", Rome, 00185, Italy
[3] La Sapienza University of Rome, Dept of Chemistry, Rome, 00185, Italy
[4] Archaeologist, Presidente FAI – Delegazione Viterbo
[5] Archaeologist
* teresa.rinaldi@uniroma1.it

a

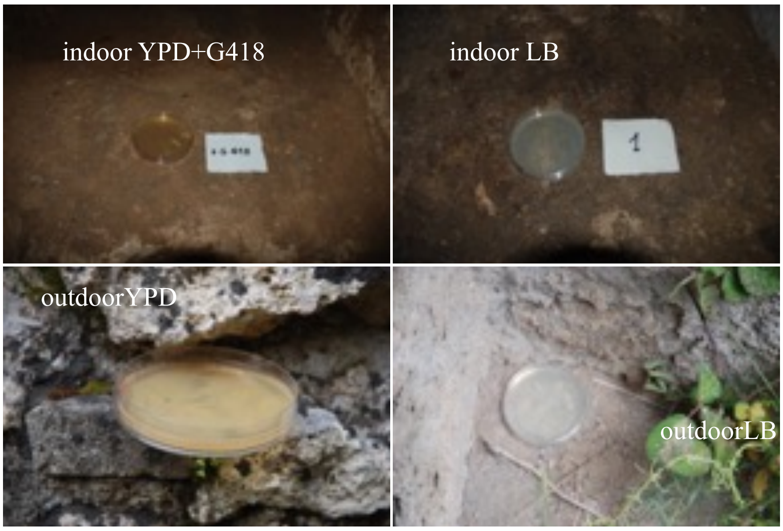

b

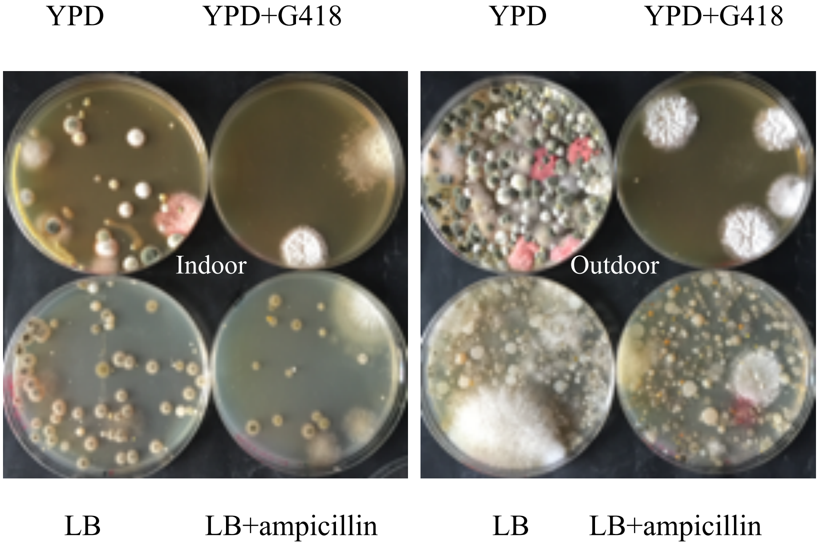

Supplementary Figure S1 Microrganisms collected from the air indoor and outdoor immediately after the opening of the tomb after sanitation. (a). As an example, 4 plates out of eight are showed during the 15 minutes left open. (b). Qualitative analysis of microrganisms collected from the air indoor (left) and outdoor (right). Plates were grown one week at 28°C. The complete media used were YPD and LB and the same media supplemented with antibiotics G418 (geneticine) or ampicillin.

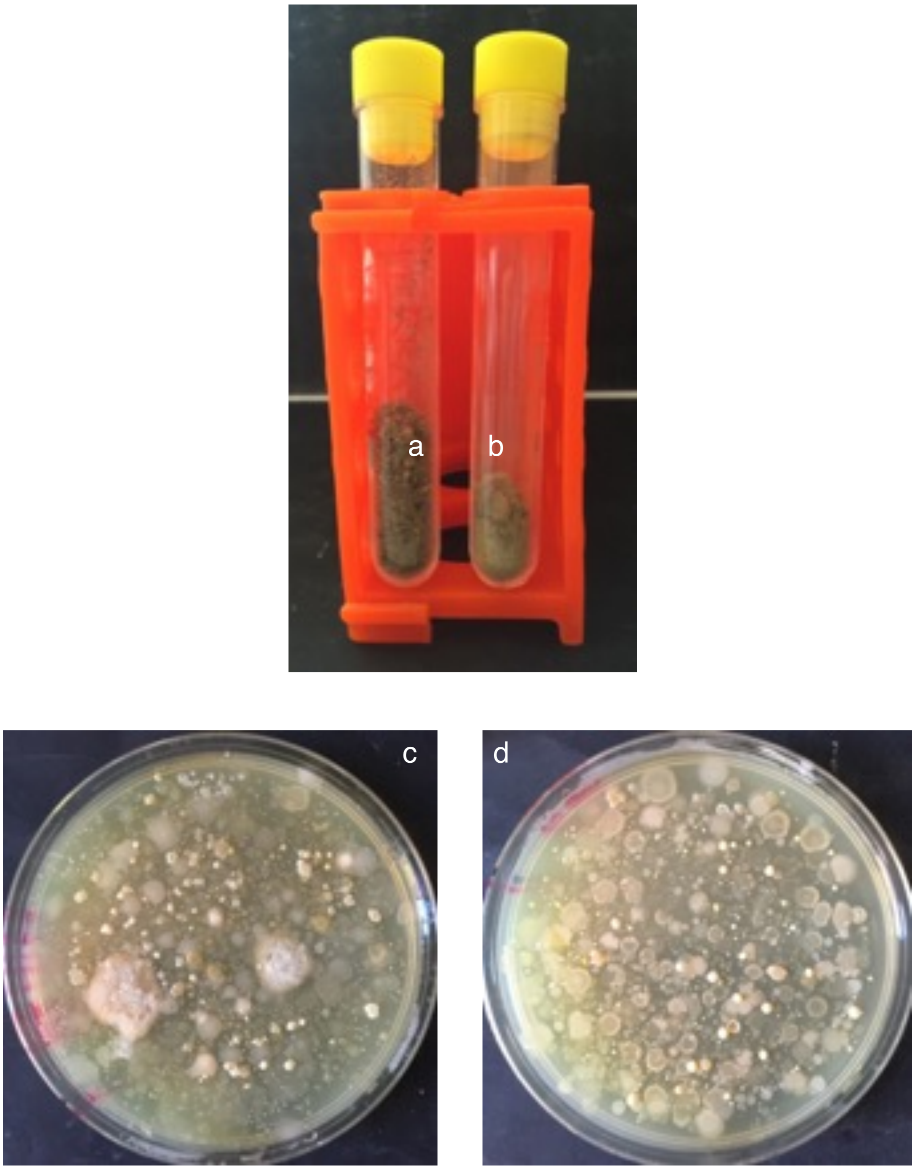

Supplementary Figure S2 Microrganisms collected from the ground. (a: sample from outdoor; b: sample from indoor). LB plates were used to plate 0,1 microgram of the samples (c: outdoor; d: indoor). Plates were grown 3 days at 28°C.

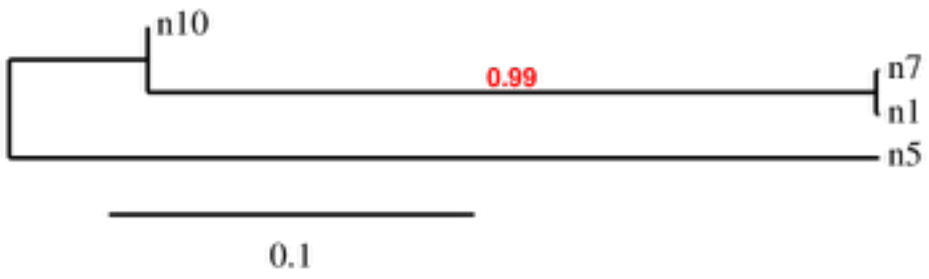

Supplementary Figure S3 Phylogenetic tree of the bacteria isolated in the *Tomba degli Scudi*. Neighbour-Joining tree based on bacterial 16S rDNA gene sequence date from four different isolates (samples 1, 5, 7, 10) along with *Escherichia coli.*

a

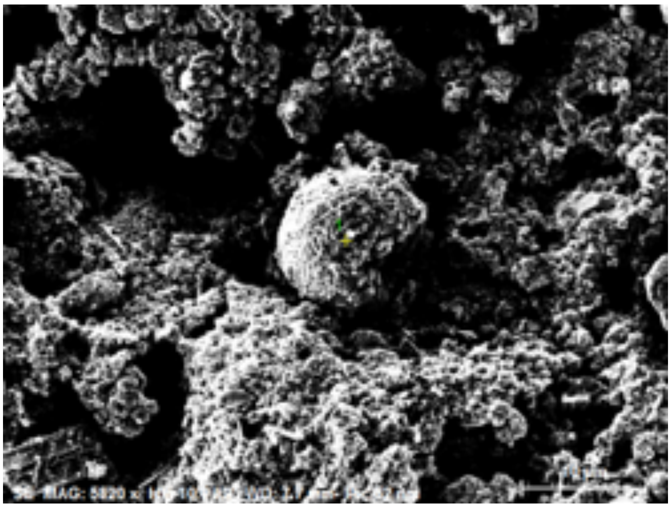

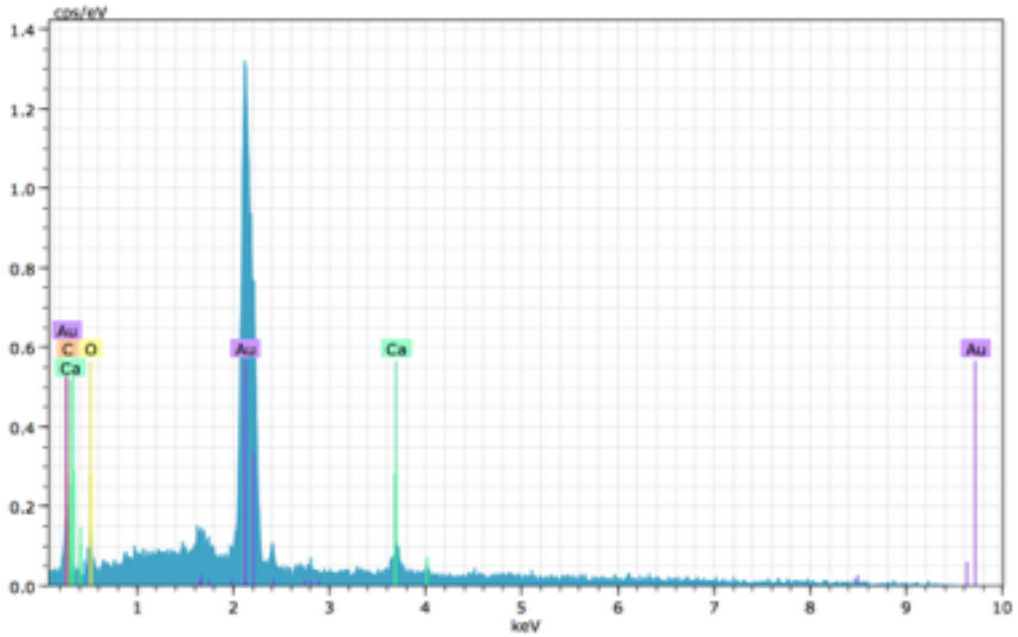

Spectrum: 1

El	AN	Series	unn. C [wt.%]	norm. C [wt.%]	Atom. C [at.%]	Error (1 Sigma) [wt.%]
O	8	K-series	1.41	38.07	45.95	0.52
C	6	K-series	0.80	21.49	34.56	0.29
Ca	20	K-series	1.50	40.44	19.49	0.12
Au	79	M-series	0.00	0.00	0.00	0.00
		Total:	3.70	100.00	100.00	

b

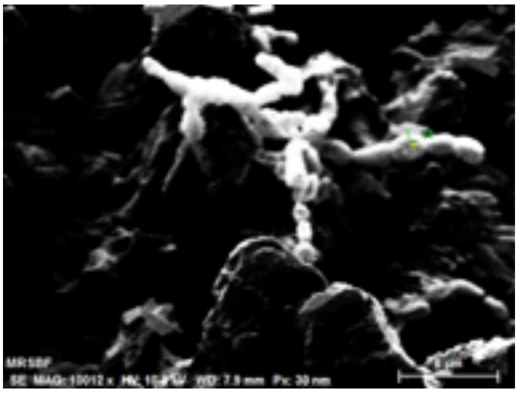

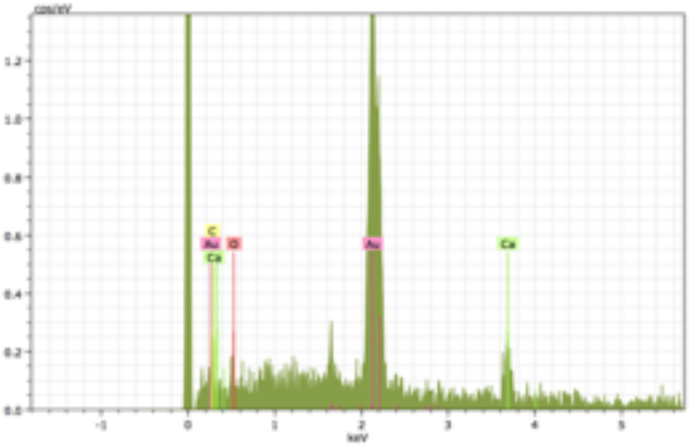

Spectrum: 1_ 11

El	AN	Series	unn. C [wt.%]	norm. C [wt.%]	Atom. C [at.%]	Error (1 Sigma) [wt.%]
O	8	K-series	1.45	38.70	55.44	0.87
Ca	20	K-series	2.03	54.18	30.98	0.22
C	6	K-series	0.27	7.12	13.58	0.26
Au	79	M-series	0.00	0.00	0.00	0.00
		Total:	3.74	100.00	100.00	

Supplementary Figure [illegible] SEM analysis of samples from eastern and western walls (a). The atomic composition of the spherical deposition in Figure 4b suggests a $CaCO_3$ composition. (b). A microorganism

present in the sample of the thick white deposition of the eastern wall (see Figure 4d) was analysed and the irregular deposition on the bacterial surfaces corresponds to $CaCO_3$.

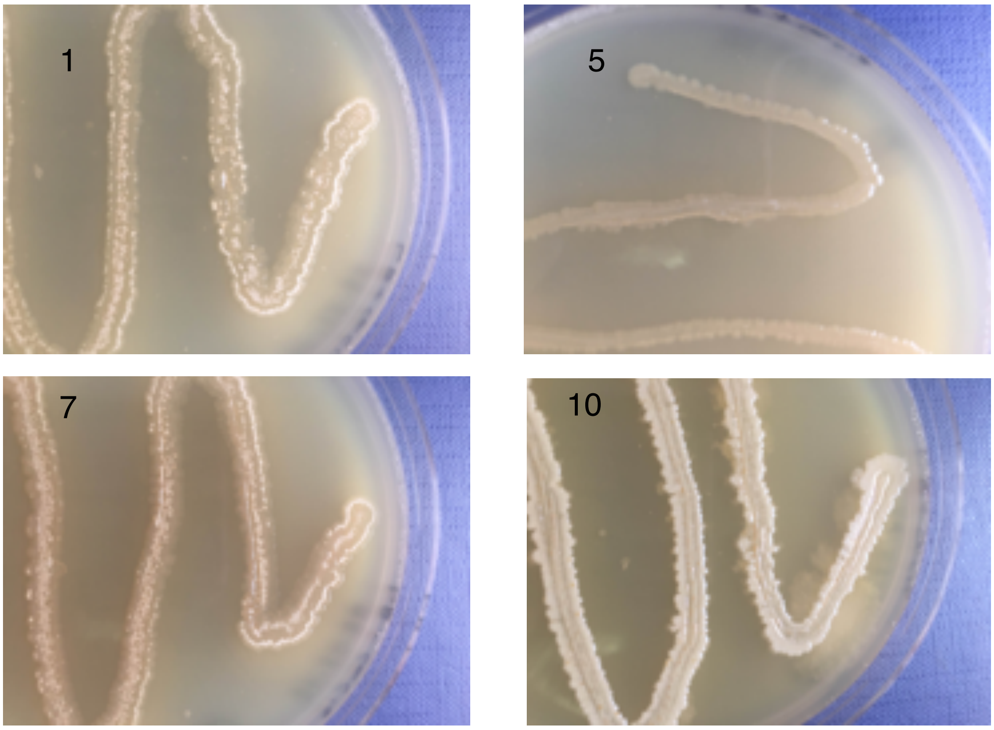

Supplementary Figure S5 Capability of the isolated *Bacillus* strains to produce calcium carbonate crystals. The strains were streaked on YPD plates containing urea and pictures were taken after a week at 28°C. Strains 1, 7 and 10 (in lower amount) showed a calcite deposition, while strain 5 was unable to produce Ca-CO_3.

Supplementary Table S1
Identification of bacterial species isolated from the walls of the *Tomba degli Scudi* based on 16S rRNA gene sequence.

Isolates	GeneBank accession number	The closest bacterial relatives	Sequence ID	Identity
1	KY283965	[Brevibacterium] frigoritolerans strain DSM 8801 16S ribosomal RNA gene, complete sequence	NR_115064.1	97%
		Bacillus simplex strain DSM 1321 16S ribosomal RNA gene, complete sequence	NR_042136.1	97%
5	KY283966	Fictibacillus barbaricus strain V2-BIII-A2 16S ribosomal RNA gene, partial sequence	NR_028967.1	96%
		Fictibacillus phosphorivorans strain Ca7 16S ribosomal RNA gene, partial sequence	NR_118455.1	96%
7	KY283967	[Brevibacterium] frigoritolerans strain DSM 8801 16S ribosomal RNA gene, complete sequence	NR_115064.1	99%
		Bacillus simplex strain LMG 11160 16S ribosomal RNA gene, partial sequence	NR_114919.1	99%
10	KY283968	Bacillus cereus ATCC 14579 16S ribosomal RNA (rrnA) gene, complete sequence	NR_074540.1	100%
		Bacillus cereus strain CCM 2010 16S ribosomal RNA gene, complete sequence	NR_115714.1	100%
